# Investigation of the Impact of the H310A FcRn Region Mutation on ^89^Zr-Immuno-PET Brain Imaging with a BBB-Shuttle Anti‑Amyloid Beta Antibody

**DOI:** 10.1007/s11307-024-01931-z

**Published:** 2024-08-02

**Authors:** Thomas E. Wuensche, Natascha Stergiou, Iris Mes, Mariska Verlaan, Esther J. M. Kooijman, Albert D. Windhorst, Allan Jensen, Ayodeji A. Asuni, Benny Bang-Andersen, Guus A. M. S. van Dongen, Danielle J. Vugts, Wissam Beaino

**Affiliations:** 1 Department Radiology & Nuclear Medicine, Amsterdam UMC Location Vrije Universiteit Amsterdam, De Boelelaan 1117, Amsterdam, The Netherlands; 2 Amsterdam Neuroscience, Brain Imaging, Amsterdam, The Netherlands; 3 H. Lundbeck A/S, Ottiliavej 9, 2500 Valby, Denmark

**Keywords:** Aducanumab, Amyloid-beta, Transferrin receptor, ^89^Zr-immuno-PET, FcRn, H310A, BBB-shuttle

## Abstract

**Purpose:**

In the emerging field of antibody treatments for neurodegenerative diseases, reliable tools are needed to evaluate new therapeutics, diagnose and select patients, monitor disease progression, and assess therapy response. Immuno-PET combines the high affinity and exceptional specificity of monoclonal antibodies with the non-invasive imaging technique positron emission tomography (PET). Its application in neurodegenerative disease brain imaging has been limited due to the marginal uptake across the blood–brain barrier (BBB). The emergence of BBB-shuttle antibodies with enhanced uptake across the BBB extended immuno-PET to brain imaging. We recently reported about specific brain uptake of a bispecific aducanumab mTfR antibody in APP/PS1 TG mice using ^89^Zr-immuno-PET. However, a sufficient target-to-background ratio was reached at a relatively late scanning time point of 7 days post-injection. To investigate if a better target-to-background ratio could be achieved earlier, an aducanumab BBB-shuttle with a mutated Fc region for reduced FcRn affinity was evaluated.

**Procedures:**

Adu^H310A^-8D3 and Adu-8D3 were modified with DFO*-NCS and subsequently radiolabeled with ^89^Zr. The potential influence of the H310A mutation, modification with DFO*-NCS, and subsequent radiolabeling on the in vitro binding to amyloid-beta and mTfR1 was investigated via amyloid-beta peptide ELISA and FACS analysis using mTfR1 transfected CHO-S cells. Blood kinetics, brain uptake, in vivo PET imaging and target engagement of radiolabeled Adu^H310A^-8D3 were evaluated and compared to non-mutated Adu-8D3 in APP/PS1 TG mice and wild-type animals as controls.

**Results:**

Radiolabeling was performed with sufficient radiochemical yields and radiochemical purity. In vitro binding to amyloid-beta and mTfR1 showed no impairment. [^89^Zr]Zr-Adu^H310A^-8D3 showed faster blood clearance and earlier differentiation of amyloid-beta-related brain uptake compared to [^89^Zr]Zr-Adu-8D3. However, only half of the brain uptake was observed for [^89^Zr]Zr-Adu^H310A^-8D3.

**Conclusions:**

Although a faster blood clearance of Adu^H310A^-8D3 was observed, it was concluded that no beneficial effects for ^89^Zr-immuno-PET imaging of brain uptake were obtained.

**Graphical Abstract:**

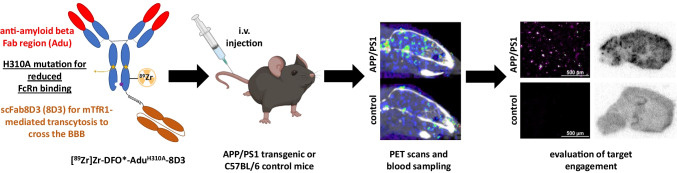

**Supplementary Information:**

The online version contains supplementary material available at 10.1007/s11307-024-01931-z.

## Introduction

With the approval of two monoclonal antibodies (mAbs), Aducanumab (ADUHELM™) and Lecanemab (LEQEMBI™), by the Food and Drug Administration (FDA) as a treatment for Alzheimer's Disease (AD), the accelerating prominence of antibody-based treatments has extended in recent years to the field of neurodegenerative diseases [1–3]. In addition to regular immunoglobulins (IgGs), bispecific antibodies fused to a transferrin receptor (TfR) binding moiety, enabling receptor-mediated transcytosis (RMT) for enhanced uptake across the blood–brain barrier (BBB) became prominent. These BBB-shuttles have shown great promise for therapeutic intervention in preclinical and clinical settings [4–6], with one anti-amyloid-beta-TfR mAb (Trontinemab) currently being evaluated in a phase II clinical trial [7, 8]. Higher brain and target-specific uptake of those BBB-shuttle mAbs were determined using the non-invasive positron emission tomography (PET) imaging technique (immuno-PET). This combination of antibodies' high affinity and exceptional specificity, receptor-mediated transcytosis, and immuno-PET opens new opportunities for diagnosis, selection of patients, monitoring of disease progression, and assessment of therapeutic response in central nervous system (CNS) disorders.

Due to their relatively slow blood clearance, regular antibodies usually lead to late imaging time points with possibly suboptimal target-to-background contrasts [9]. Furthermore, slow kinetics lead to higher radiation doses for patients and limits the use of PET radionuclides with matching half-lives like ^89^Zr (t_1/2_ = 3.26 d) and ^124^I (t_1/2_ = 4.18 d). Hence, achieving a faster blood clearance could be beneficial to advance brain immuno-PET further into clinical applications, especially for patient selection.

Several strategies for constructs utilizing mTfR1-mediated transcytosis have been pursued to increase blood clearance, such as employing smaller constructs like affibodies or di-scFv's, or using clearing agents to remove the antibody from the blood circulation [10–13]. Another appealing approach, successfully applied for peripheral imaging applications, is the mutation of the antibody Fc domain to alter its affinity to the neonatal Fc receptor (FcRn). With the mutation of a crucial amino acid for the Fc-FcRn interaction, His310Ala (H310A), in the C_H_2 domain (EU numbering system), the protective role from lysosomal degradation is reduced. Hence, the serum persistence of antibody constructs is decreased significantly [14, 15].

We recently demonstrated amyloid-beta imaging with ^89^Zr-labeled aducanumab BBB-shuttle antibody. This mAb was modified for mTfR1-mediated transcytosis via a fused anti-transferrin receptor binding single chain Fab fragment derived from 8D3 (Adu-8D3) [16, 17]. With this ^89^Zr-labeled BBB-shuttle, a sufficient target-to-background ratio contrast was observed at a relatively late scanning time point of 7 days post-injection (p.i.). To investigate the possibility of allowing earlier imaging time points with potentially higher target-to-background ratios, the Adu-8D3 was modified with the H310A mutation to reduce binding to the FcRn. The blood kinetics, brain uptake, and target engagement of ^89^Zr-labeled Adu^H310A^-8D3 were investigated *ex vivo* and *in vivo* by PET imaging in APP/PS1 TG mice and C57BL/6 WT mice (from here on referred to as WT control mice) as control. In addition, it was compared to non-mutated Adu-8D3 in APP/PS1 TG mice.

## Methods

### General materials

1 M HEPES (pH 7.0—7.5) was obtained from Invitrogen, and phosphate-buffered saline was obtained from Brunschwig Chemie. ^89^Zr in 1 mol/L oxalic acid was obtained from Perkin-Elmer (Boston, USA). Water was distilled and deionized using a MilliQ water filtration system (Millipore, USA). DFO*-NCS was synthesized by Mercachem B.V. (Nijmegen, The Netherlands). The ELISA antigen, Var24 (amyloid-beta vaccine construct consisting of three amyloid-beta 1–12 peptides), was provided by H. Lundbeck A/S, Valby, Denmark. All other starting reagents and solvents were obtained from Merck/Millipore Sigma.

## Antibody Constructs

All antibodies were produced by H. Lundbeck A/S, Valby, Denmark.

### Antibody Design

All antibodies are built on the human IgG1 framework and hold Fc-null mutations K322A, L234A, and L235A (LALAKA), as described by Lin et al. and Hezareh et al. to reduce binding to FcγR [18, 19]. The bispecific mAbs with monospecific murine TfR1 binding (scFab-8D3) were generated using the Knobs-in-Hole (KIH) technology [20]. On the Knob heavy chain (HC) LSPG termini, a GGS(GGGGS)_3_ linker followed by the 8D3 scFab extension (chain separated by a (GGGGS)_6_ linker) was engineered at the C-terminal. The Hole HC has a typical Fc length. In the case of Adu^H310A^-8D3, the Knob and Hole HC's hold the H310A to reduce binding to FcRn. B12^H310A^-8D3 (B12 targets gp120 of HIV1) was designed similarly to Adu^H310A^-8D3 and used as a control antibody to assess non-specific uptake.

### Cell Culture

Synthetic genes of the HC and light chain (LC) optimized with human codon composition were sub-cloned into the pTT5 vector for transient expression. Transfection of HC and LC expression vectors was performed in HEK293 6E cells using PEIpro (Polyplus) as a transfection reagent. The HEK293 6E expression system, including the pTT5 vector, is licensed from the National Research Council of Canada (NRCC). Transfected cells were cultured until the viability had dropped to around 50%, and culture media was harvested by centrifugation and sterile filtration and kept cold (4 °C) until purification.

### Purification

Antibodies in the cell culture harvest were purified by capturing on a HiTrap protein G (Cytiva), followed by washing with PBS and elution with 0.1 M Glycine pH 2.7. After dialysis against 20 mM Tris pH 7.5, the sample was passed through a Q-sepharose column (Cytiva) and equilibrated with the same buffer. The flow-through was concentrated to < 5 mL and purified on a HiLoad® 16/600 Superdex® (Cytiva) using PBS as eluent. Fractions were analyzed by SDS-PAGE, SEC, and LC–MS. Selection for pooling was made to minimize aggregates, incorrectly paired molecules, and free LC. After purification, a certain amount of Hole dimer (double Hole HC with typical Fc length) was still present in the used Adu^H310A^-8D3 and B12^H310A^-8D3 batches (Figure S3 and S4; t_R_ = 15.2/16.3; ~ 20%).

## Synthesis of ^89^Zr-Labeled Compounds

### DFO*-Adu^H310A^-8D3, DFO*-B12^H310A^-8D3 and DFO*-Adu-8D3

DFO*-Adu^H310A^-8D3, DFO*-B12^H310A^-8D3, and DFO*-Adu-8D3 were prepared as described previously [17]. 1 mg of the antibody was diluted to 4 mg/mL with 0.9% NaCl, which resulted in a total volume of 250 µL. The pH was adjusted to 8.9–9.1 with 0.1 M Na_2_CO_3_ and added to DFO*-NCS solution in DMSO (10 µL, 5 mM, 10 eq.). Immediate resuspension with the pipette was performed to ensure rapid mixing of the reaction solution. Afterward, the reaction mixture was incubated in a ThermoMixer™ at 37 °C and 550 rpm for 2 h. At the end of the incubation, the reaction mixture was applied on a PD-10 column (GE Healthcare Life Sciences), and fractions of 0.5 mL were collected using 50 mM sodium acetate/200 mM sucrose + 0.01% Tween-20, pH = 5.4–5.6 (from now on referred as formulation buffer). Each fraction was measured on a NanoDrop™ spectrophotometer (Thermo Fisher); fractions with the highest UV absorbance at 280 nm were pooled, and the concentration was determined via SE-HPLC using a calibration curve of the respective antibody. Shortly before the radiolabeling, a concentration and buffer exchange (0.5 M HEPES) step was performed via spin filtration.

### ^89^Zr-Labeling

All DFO*-conjugated mAbs were radiolabeled as described previously [17]. 150–250 MBq [^89^Zr]Zr-oxalate in 1 M oxalic acid solution was pipetted into a 1.5 mL Eppendorf vial, and 1 M oxalic acid was added to reach a volume of 150 µL. Next, 67.5 µL 2 M Na_2_CO_3_ were added and reacted for 3 min. Subsequently, 375 µL 1 M HEPES buffer was added. Afterward, 500 µg of the modified mAb in 0.5 M Hepes were added to the reaction mixture and incubated in a ThermoMixer™ (Eppendorf) at room temperature (RT) and 550 rpm for 1 h. The reaction mixture was then applied to a PD-10 column, and fractions of 0.5 mL were collected by eluting with formulation buffer. Each fraction was measured with a dose calibrator (Veenstra Instruments); fractions with the highest activity were pooled, and the concentration was determined via SE-HPLC. The yield of the radiolabelling was calculated by the following formula:

Radiochemical yield = Activity_radiolabeled antibody_ x radiochemical purity/Activity_total_ × 100%

Unlabeled mAb and formulation buffer were added to formulate the product to 30 µg mAb with a specific activity of 0.19 to 0.21 MBq/µg mAb in ~ 150 µL formulation buffer per mouse.

## Quality Controls

### Radiochemical Purity, Antibody Concentration, Antibody Integrity, And Antigen-Binding

Radioimmunoconjugates were checked for radiochemical purity by spin filter analysis following a described procedure [21]. Antibody concentration and integrity were determined by SE-HPLC; detailed information and chromatograms are given in the supplementary information. Antigen binding to amyloid-beta and mTfR1 was evaluated as described previously [17]. Detailed information and results are provided in the supplementary information.

## *In vivo* Experiments

Animal experiments were performed according to the NIH Principles of Laboratory Animal Care, the European Community Council Directive (2010/63/EU) for laboratory animal care, and the Dutch Law on animal experimentation ("Wet op de dierproeven," Stb 1985, 336). The experimental protocol was validated and approved by the central Dutch national committee for animal experimentation (CCD) and the local committee on animal experimentation of the Amsterdam UMC, Vrije Universiteit Amsterdam. The transgenic C57BL/6 J-Tg(Thy1-APPSw-Thy1-PSEN1*L166P)21/Jckr, designated in this paper as APP/PS1 TG mice, carry a transgene insertion for the human Abeta42 [22]. The female or male APP/PS1 mice and the wild-type control mice (received at 10 to 12 months old from Charles River) were left for at least one week of acclimatization before starting experiments.

## Biodistribution

The biodistribution of the radiolabeled mAbs ([^89^Zr]Zr-Adu^H310A^-8D3 and [^89^Zr]Zr-B12^H310A^-8D3) constructs was determined as follows: 30 µg radiolabeled antibody in 130–170 µL formulation buffer were injected intravenously into the tail vein under anesthesia with inhalation of 2–4% isoflurane in oxygen. Biodistribution was determined at 24 and 72 h p.i.. Blood and organs of interest were collected and weighed for all mice, and the amount of radioactivity in each sample was measured in a gamma counter (LKB Wallac Gamma Counter, model 1282 Compugamma CS). The brain was dissected into two hemispheres, using the left cerebral hemisphere to measure brain uptake, while the right cerebral hemisphere was used for immunohistochemistry and autoradiography. The radioactive uptake was calculated as the percentage of injected dose per gram of tissue (%ID/g), subtracting the uptake in the tail from the total amount of injected activity.

## *Ex vivo* Autoradiography

During animal dissection, the right cerebral hemisphere was flash-frozen in isopentane at -30 °C. A cryostat-microtome was used to cut the frozen right mouse brain hemispheres into 20 µm sections, which were mounted on gelatinized glass slides. Sections were exposed for 2 weeks on a phosphor screen BAS-IP SR 2040 E (General Electric, Eindhoven, the Netherlands). After exposure, the plates were scanned using a Typhoon FLA 7000 imager (General Electric, Eindhoven, the Netherlands).

## Immunofluorescence Staining

After the exposure, the same sagittal sections used for autoradiography were fixed in cold acetone (approx. -15 °C), quickly dried under a fan, and blocked with 20% normal goat serum for 1 h at RT. The tissues were then incubated with a Goat anti-human IgG [H + L] Cross-Adsorbed Secondary HRP-Antibody (Invitrogen; 0.4 µg/mL, 1:2000) at RT for 1 h under dark conditions. The tissues were washed 3 × 5 min with 0.05% Tween-20 in PBS, followed by a final wash step with deionized water (dH_2_O) for 5 min. Subsequently, the tissues were incubated with a 0.125% freshly filtered Thioflavin S solution at RT for 8 min under dark conditions. Then, the tissues were washed for 3 min in each of the following solutions: 2 × 80% EtOH, 1 × 90% EtOH, 3 × dH_2_O. The tissues were mounted with ProLong™ Gold Antifade Mountant (Invitrogen™, P36930). Images of the stained sections were taken with a fluorescence microscope (Zeiss Axio Observer with a Colibri 7 LED light source and an Axiocam 506 monochrome camera) and equally processed using the Zen blue software Version 3.4.

## PET Imaging

The PET imaging was performed with dedicated small animal NanoPET/CT and NanoPET/MR scanners (Mediso Ltd., Hungary) equipped with identical PET components. Mice were anesthetized by inhaling 2–4% isoflurane in oxygen (1 L/min) during the entire scanning period. PET scans were acquired for 60 min. A 5-min CT scan was acquired prior to each PET scan and used for attenuation and scatter correction purposes. Reconstruction was performed using a 3-dimensional reconstruction algorithm (Tera-Tomo; Mediso Ltd.) with four iterations and six subsets, resulting in an isotropic 0.4-mm voxel dimension and attenuation and scatter correction. Radioactivity uptake was calculated as the percentage of the injected dose per gram of tissue (%ID/g) with the decay-corrected amount of injected radiolabeled compound. Images were analyzed and quantified using the VivoQuant software (Invicro, Boston, USA), and regions of interest (ROI) were applied using the VivoQuant-integrated brain atlas tool. An example ROI is given in Figure S11.

## Statistics

Statistical analysis was performed on the brain uptake values of the different groups of mice with the Welch's t-test. Both assume normal Gaussian distribution of the values and do not assume equal variances between groups. Two-sided significance levels were calculated, and p < 0.05 was considered to be statistically significant. All graphs were generated using GraphPad Prism 9.10 software.

## Results

### Antibodies, ^89^Zr-Labeling, and in vitro Binding

**Fig. 1:**
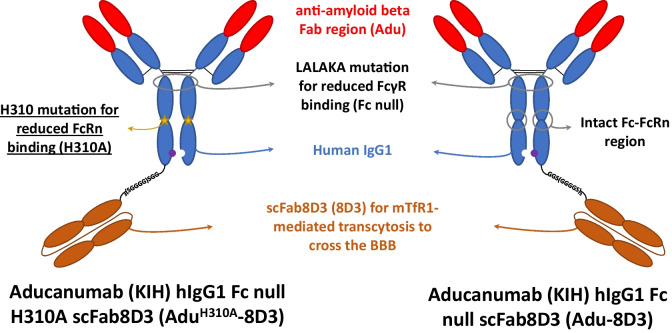
Overview of the two bispecific antibodies used in this study.

The aducanumab derived antibodies were built on the human IgG1 framework and hold Fc-null mutations L234A, L235A, and K322A (LALAKA) to reduce binding to FcγR [18, 19]. Both antibodies bind insoluble and soluble aggregates, e.g., protofibrils/oligomers, but not amyloid-beta monomers [23]. The specific binding to mTfR1 was introduced by the scFab8D3 extension via a linker on the C-terminal of the heavy chain (HC). Finally, Adu^H310A^-8D3 has the H310A mutation to reduce binding to the FcRn region, resulting in a reduced serum half-life (Fig. 1, left) [14, 15].

The potential influence of the H310A mutation on the in vitro binding to amyloid-beta and mTfR1 was investigated via amyloid-beta peptide ELISA and FACS analysis using mTfR1 transfected CHO-S cells. No impaired binding to amyloid-beta and mTfR1 for Adu^H310A^-8D3 compared to Adu-8D3 was observed (Figure S1, Table S2). The modification with DFO*-NCS and subsequent radiolabeling with ^89^Zr were performed as previously reported with sufficient radiochemical yields (82—95%) and radiochemical purity (≥ 97%) (Table S1) [16, 17]. The *in vitro* binding of the radiolabeled Adu^H310A^-8D3 and Adu-8D3 to amyloid-beta and of the DFO*-modified constructs to mTfr1 showed no impairment (Figure S1, Table S2). These results show the inertness of introducing the H310A mutation and the modification with DFO*-NCS and subsequent radiolabeling.

## Blood Kinetics of [^89^Zr]Zr-Adu^H310A^-8D3 and [^89^Zr]Zr-Adu-8D3 in Mice

**Fig. 2:**
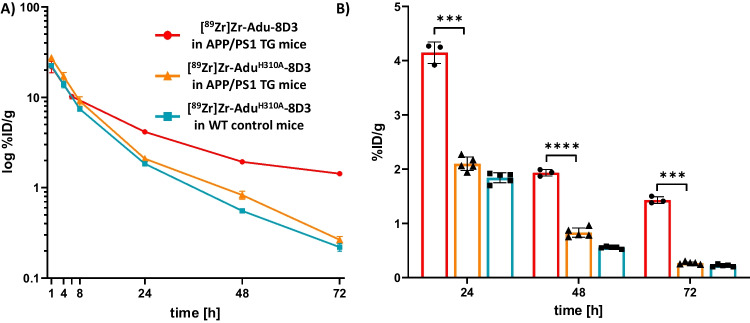
Blood kinetics of [^89^Zr]Zr-Adu^H310A^-8D3 in APP/PS1 TG and WT control mice as well as blood kinetics of [^89^Zr]Zr-Adu-8D3 in APP/PS1 TG mice. 10–12 months old APP/PS1 TG or WT control mice were injected with 1 mg/kg radioimmunoconjugates, and blood sampling was performed up to 72 h p.i. (**A**) Blood kinetics of all three groups (**B**) Bar graph of the blood levels at 24, 48, and 72 h p.i

To evaluate the effect of the H310A mutation on the blood kinetics, [^89^Zr]Zr-Adu^H310A^-8D3 and [^89^Zr]Zr-Adu-8D3 were i.v. injected into APP/PS1 TG mice, and the blood kinetics were determined up to 72 h post-injection (p.i.) (Fig. 2). A rapid drop in radioactivity was observed for both [^89^Zr]Zr-Adu^H310A^-8D3 and [^89^Zr]Zr-Adu-8D3 between 1 and 8 h p.i.. At 24 h p.i. for [^89^Zr]Zr-Adu^H310A^-8D3, a significantly faster blood clearance was observed compared with [^89^Zr]Zr-Adu-8D3 (2.1 ± 0.1%ID/g and 4.2 ± 0.2%ID/g, respectively). This difference was also observed at 72 h p.i. (0.2%ID/g and 1.4 ± 0.1%ID/g, respectively). In WT control mice, [^89^Zr]Zr-Adu^H310A^-8D3 showed similar blood kinetics as in APP/PS1 TG mice. The non-logarithmic presentation of the blood kinetics is given in Figure S7.

## Brain Uptake Kinetics of [^89^Zr]Zr-Adu^H310A^-8D3 and [^89^Zr]Zr-Adu-8D3 in Mice

**Fig. 3:**
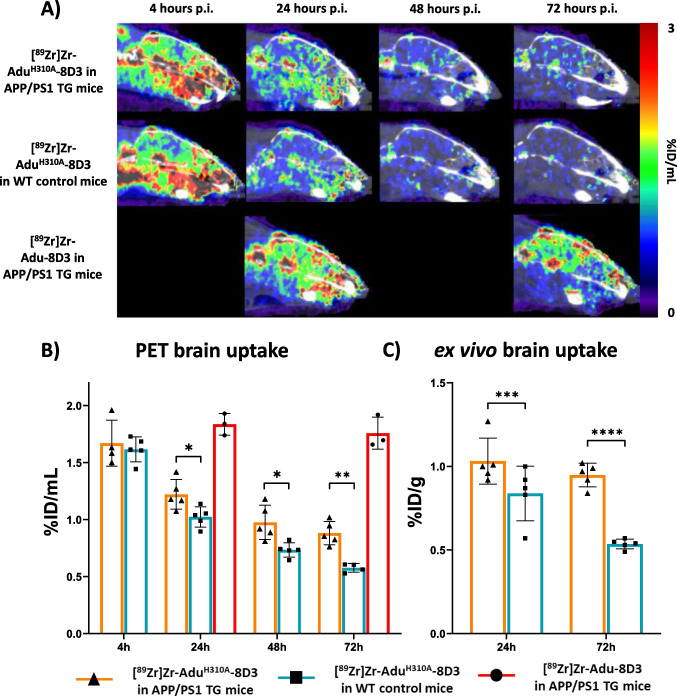
Brain uptake kinetics of [^89^Zr]Zr-Adu^H310A^-8D3 in APP/PS1 TG and WT control mice and [^89^Zr]Zr-Adu-8D3 in APP/PS1 TG mice. 10–12 month old APP/PS1 TG or WT control mice were injected with 1 mg/kg radioimmunoconjugates and imaged with PET/CT and PET/MRI between 4 and 72 h p.i. Ex vivo analysis was performed at 24 and 72 h p.i. for [^89^Zr]Zr-Adu^H310A^-8D3 in APP/PS1 TG and WT control mice (**A**) One sagittal representative PET/CT image is shown per group. (**B**) PET brain uptake quantification. (**C**) Ex vivo brain uptake of [^89^Zr]Zr-Adu.^H310A^-8D3 in APP/PS1 TG and WT control mice. Significant differences between the groups are marked with asterisks (*p < 0.1; **p < 0.01; ***p < 0.001; ****p < 0.0001).

To investigate the performance of [^89^Zr]Zr-Adu^H310A^-8D3 for visualizing amyloid-beta in the brain and the time point for the optimal imaging contrast, PET imaging in APP/PS1 TG and WT control mice was performed at 4 h, 24, 48, and 72 h p.i. (Fig. 3A). Brain uptake was quantified at different time points and compared (Fig. 3B). At 4 h p.i. brain uptake of [^89^Zr]Zr-Adu^H310A^-8D3 in APP/PS1 TG and WT control mice was similar with 1.67 ± 0.2%ID/mL and 1.62 ± 0.11%ID/mL, respectively. At 24 and 48 h p.i., a significant difference between the brain uptake of [^89^Zr]Zr-Adu^H310A^-8D3 in APP/PS1 mice (1.22 ± 0.13%ID/mL 24 h p.i.; 0.97 ± 0.15%ID/mL 48 h p.i.) compared to WT mice (1.02 ± 0.09%ID/mL 24 h p.i.; 0.73 ± 0.06%ID/mL 48 h p.i.) was observed. At 72 h p.i., an even higher significance difference in uptake was observed. This higher uptake of [^89^Zr]Zr-Adu^H310A^-8D3 in APP/PS1 TG mice indicates an amyloid-beta-specific target engagement of the antibody, leading to its higher retention in the brain while the control group displays a gradual washout. [^89^Zr]Zr-Adu-8D3 showed significantly higher PET brain uptake than [^89^Zr]Zr-Adu^H310A^-8D3 at 24 h p.i. (1.84 ± 0.1%ID/mL) and twofold higher brain uptake at 72 h p.i. (1.76 ± 0.14%ID/mL) in APP/PS1 TG mice. An H310A mutated version of the bifunctional anti-HIV-monoclonal antibody, [^89^Zr]Zr-B12^H310A^-8D3, was used as isotype control in APP/PS1 mice. However, PET imaging data could not be compared due to the relatively slow kinetics of the [^89^Zr]Zr-B12^H310A^-8D3 compared to [^89^Zr]Zr-Adu^H310A^-8D3, which resulted in slow blood clearance and higher non-specific brain uptake (Figure S8).

## *Ex vivo* Biodistribution of [^89^Zr]Zr-Adu^H310A^-8D3 in Mice

**Fig. 4:**
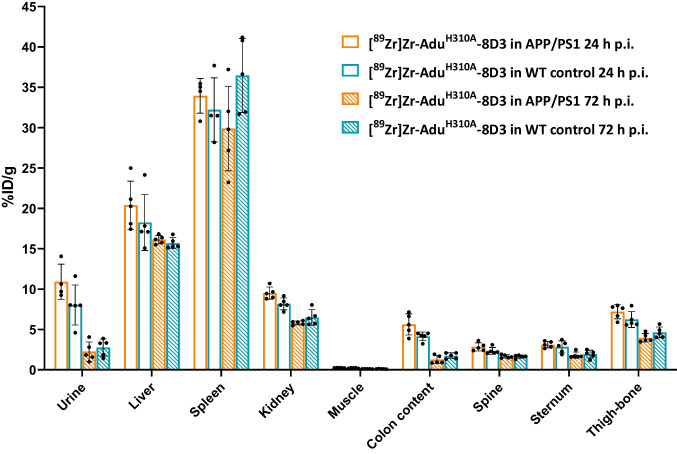
*Ex vivo* biodistribution of [^89^Zr]Zr-Adu.^H310A^-8D3 in APP/PS1 TG and WT control mice. 10 months old APP/PS1 TG or WT control mice were injected with 1 mg/kg radioimmunoconjugate. Ex vivo analysis was performed at 24 and 72 h p.i.. Data is expressed as percent injected dose per gram tissue (%ID/g).

To validate the PET imaging results, *ex vivo* biodistribution of [^89^Zr]Zr-Adu^H310A^-8D3 in APP/PS1 TG and WT control mice at 24 h and 72 h p.i. was determined (Figs. 3C and 4). Biodistribution with [^89^Zr]Zr-Adu-8D3 in the APP/PS1 TG and WT control mice was previously reported and, therefore, not repeated in this study [16]. At 24 h p.i., a significantly higher brain uptake of [^89^Zr]Zr-Adu^H310A^-8D3 in APP/PS1 TG mice (1.03 ± 0.14%ID/g) compared to WT control mice (0.84 ± 0.16%ID/g) was observed. At 72 h p.i., the difference in brain uptake increased further with 0.95 ± 0.07%ID/g in APP/PS1 mice compared to 0.54 ± 0.03%ID/g in WT control mice (Fig. 3C). Notably, while brain uptake of [^89^Zr]Zr-Adu^H310A^-8D3 in WT control mice decreased steadily, brain uptake of [^89^Zr]Zr-Adu^H310A^-8D3 in APP/PS1 TG mice exhibited a slower rate of decrease over time. These findings align with the PET results (Fig. 3B). Biodistribution of the peripheral organs showed a similar pattern as previously reported for [^89^Zr]Zr-Adu-8D3 at day 3 p.i. (Fig. 4, Table S3-4) [16]. High uptake was observed in the spleen, followed by the catabolic organs, such as the kidney and liver. Modest uptake in bone, bone marrow, and spinal cord was observed, and there was no relevant uptake in muscle tissue. In WT control mice, [^89^Zr]Zr-Adu^H310A^-8D3 showed similar *ex vivo* biodistribution as in APP/PS1 mice (Fig. 4).

## Evaluation of Target Engagement

**Fig. 5:**
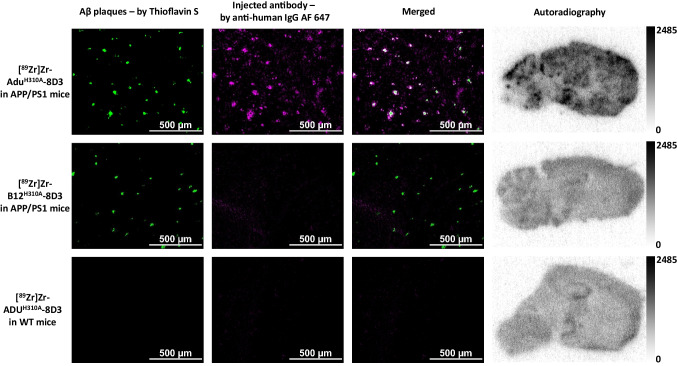
Ex vivo autoradiography and immunofluorescence analysis of 20 μm brain cryo-sections of APP/PS1 TG and WT control mice at 72 h p.i.. 10 month old APP/PS1 TG and WT control mice were injected with 1 mg/kg radiolabeled antibody. The exposure time for autoradiography was 2 weeks. The same sections were stained with 0.125% Thioflavin S (green) and AF647-goat anti-human IgG (1:1000, purple) to detect the injected antibody. The images of each separate and merged channel are shown; the overlay of the two signals appears in white.

To validate that the higher brain uptake of [^89^Zr]Zr-Adu^H310A^-8D3 in APP/PS1 TG mice compared to WT mice is due to the target engagement with amyloid-beta, *ex vivo* autoradiography and immunofluorescence staining on brain tissue post-imaging 72 h p.i. was performed. *Ex vivo* autoradiography showed a granular uptake pattern of radioactivity, similar to plaque distribution, for [^89^Zr]Zr-Adu^H310A^-8D3 in APP/PS1 TG mice only and not in WT control mice (Fig. 5). Additional immunostaining was performed on the same tissue using Thioflavin S to detect amyloid-beta plaques and a secondary anti-Human-IgG to detect the injected bispecific antibody. The injected [^89^Zr]Zr-Adu^H310A^-8D3 co-localized with amyloid-beta. In WT control mice, no amyloid-beta plaques or [^89^Zr]Zr-Adu^H310A^-8D3 were detected (Fig. 5). A magnified version with zoom-in on fibrils is given in Figure S9. The same experiments were performed on the brains of APP/PS1 TG mice injected with [^89^Zr]Zr-B12^H310A^-8D3 as a control. No specific uptake was observed by autoradiography, and no injected antibody was detected in the brain (Fig. 5). Thus, *ex vivo* immunofluorescence staining aligns with the findings of the autoradiography, PET imaging data, and *ex vivo* brain uptake, showing amyloid beta-specific brain uptake only for [^89^Zr]Zr-Adu^H310A^-8D3 in APP/PS1 TG mice.

## Discussion

With the emerging field of antibody treatment for neurodegenerative diseases, reliable tools are needed to evaluate new therapeutics, diagnose and select patients, monitor disease progression, and assess therapy response. Immuno-PET combines the high affinity and exceptional specificity of monoclonal antibodies with the non-invasive PET imaging technique. The application in neurodegenerative disease brain imaging is limited, most probably due to the limited uptake across the BBB. In recent years, the emergence of brain-shuttle antibodies with enhanced uptake across the blood–brain barrier extended the use of ^124^I-immuno-PET to brain imaging [24]. We established ^89^Zr-immuno-PET to pair these BBB-shuttle constructs with a PET radionuclide with superior imaging qualities and better availability than ^124^I [16, 17, 25, 26]. These studies revealed that the optimal timepoint for PET imaging with ^89^Zr-labeled bispecific aducanumab (Adu-8D3), targeting amyloid-beta plaques and mTfR1 for BBB shuttling, was 7 days p.i.. To investigate if a better target-to-background ratio can be achieved earlier, a faster clearing BBB-shuttle antibody having a His310Ala mutation (Adu^H310A^-8D3) to reduce FcRn binding was evaluated in APP/PS1 TG mice.

Evaluation of the blood kinetics revealed a faster clearance for [^89^Zr]Zr-Adu^H310A^-8D3 compared to the recently reported [^89^Zr]Zr-Adu-8D3 [16]. The faster blood clearance resulted, at an earlier time point, in a significantly higher brain uptake in APP/PS1 TG mice compared to WT control mice. For [^89^Zr]Zr-Adu^H310A^-8D3, this was at 24 h p.i. while for [ ^89^Zr]Zr-Adu-8D3, this was at 72 h p.i. as previously reported [16, 17]. However, in both cases, the contrast enhanced further over time, leading to a more favorable target-to-background ratio at 72 h p.i. for [^89^Zr]Zr-Adu^H310A^-8D3 in APP/PS1 TG mice compared to WT control mice. Since the same trend was reported for [^89^Zr]Zr-Adu-8D3 (168 h versus 72 h p.i.), it can be concluded that in addition to blood activity levels, the slower efflux of amyloid-beta-related radiotracer uptake also plays a key role in reaching optimal imaging contrast. The isotype control B12^H310A^-8D3 in APP/PS1 TG mice showed a similar trend as Adu^H310A^-8D3 in WT control mice but was retained longer in the blood (Figure S7). Therefore, brain uptake levels were overall higher, and B12^H310A^-8D3 was not suitable to be directly compared to Adu^H310A^-8D3. The specific amyloid-beta binding of Adu^H310A^-8D3 was confirmed by *ex vivo* immunofluorescence and autoradiography. None or negligible antibody was detectable for B12^H310A^-8D3 in APP/PS1 TG mice, which is in line with what was previously reported for [^89^Zr]Zr-B12-8D3 in APP/PS1 TG mice (Fig. 5) [17]. A comparable biodistribution pattern (except the brain) was observed for [^89^Zr]Zr-Adu^H310A^-8D3 and [^89^Zr]Zr-Adu-8D3 in APP/PS1 TG and WT control mice. High spleen uptake can be related to the high TfR expression in this organ and its function to salvage red blood cells [27]. Uptake in the kidney and liver is related to their function as catabolic organs. At the same time, the uptake in bone-related tissue can be explained by the tropism of ^89^Zr to bones and the TfR-expressing erythrocyte progenitor cells in the bone marrow [28].

Although an earlier amyloid-beta-related imaging time point through a reduced plasma-half life was achieved, a substantial downside is the overall lower brain uptake of [^89^Zr]Zr-Adu^H310A^-8D3 with only ~ 1%ID/g at 24 and 72 h p.i., which is about half the brain uptake of [^89^Zr]Zr-Adu-8D3. Furthermore, the difference in amyloid-beta driven uptake in APP/PS1 TG versus WT control mice is smaller: only 1.2 fold higher for [^89^Zr]Zr-Adu^H310A^-8D3 versus 2.2 fold higher for [^89^Zr]Zr-Adu-8D3 as reported previously [16]. This makes the H310A mutation less favorable in ^89^Zr-immuno-PET applications for CNS targets since the inferior capability to differentiate between disease and healthy control group could translate to limited visualization of specific target uptake in earlier stages of neurodegenerative diseases, which is a crucial aspect in current CNS PET tracer development. Putting these findings in context to similar work, smaller bispecific constructs with mTfR1-mediated brain shuttling and no Fc region also achieved a faster blood clearance [10, 11, 13]. In these studies, more rapid blood clearance resulted in lower amyloid-beta-specific brain uptake. This aligns with past findings, which demonstrated that a longer serum half-life with more prolonged circulation would increase the interactions with the TfR receptor on the BBB, resulting in better brain drug delivery [29]. Notable, in some cases, altered *in vitro* affinities to mTfR1 or amyloid-beta were observed compared to the native construct. In this work, the H310A mutation and the DFO* modification with the subsequent ^89^Zr-radiolabeling did not alter the *in vitro* affinity to mTfR1 or amyloid-beta of [^89^Zr]Zr-Adu^H310A^-8D3 when compared to Adu-8D3 (Figure S1; Table S2).

During the time these studies were conducted, it was considered that transcytosis across the BBB is independent of direct FcRn interactions, as was shown by Selin et al. by comparing ^125^I-labelled 8D3 and a Fab fragment of 8D3 (Fab-8D3), which lacks the Fc fragment [24]. These findings align with earlier studies for regular IgGs, stating that the limited antibody uptake across the BBB occurs independent of FcRn interactions [30–32]. In contrast, several groups have concluded that the Fc-FcRn interaction does influence mAb uptake across the BBB and the efflux of mAbs out of the brain [33–35]. However, the experimental setups of these studies differ significantly (e.g., animal models, route of mAb delivery, methods of uptake quantification, and dosing) from this study and the one from Selin et al. Therefore, we conclude that while the Fc-FcRn interaction may play a role in mAb brain uptake and efflux, the impact of mTfR binding potentially outweighs these effects.

Despite the less favorable results regarding the effect of Fc-FcRn mutations on ^89^Zr-immuno-PET in CNS applications, these mutations could be promising for applying other radionuclides with less or non-residualizing behavior. For ^124^I (t_1/2_ = 4.18 d), a non-residualizing PET radionuclide, earlier time points 3 days post-injection were achieved with an intact Fc-FcRn interaction in the past due to significantly lower non-amyloid beta related brain uptake [24]. However, there are limitations when using ^124^I, such as availability, price, and imaging quality [25, 26]. The clinically relevant SPECT radionuclide ^123^I (t_1/2_ = 13.2 h) could be a suitable alternative. Despite the lower spatial resolution of this imaging technique in clinical settings, a small molecule anti-amyloid-beta SPECT radiotracer, ^123^I-ABC577, was able to differentiate Alzheimer’s disease patients from healthy controls [36]. For immuno-SPECT applications, significant brain retention of RmAb158-scFv8D3 was shown in tg-ArcSwe mice at day 3 p.i. even with the unfavorable SPECT radionuclide ^125^I [37]. These considerations suggest that brain-penetrating antibodies with faster blood clearance and the favorable SPECT radionuclide ^123^I could be a promising approach for CNS applications. In addition, using the H310A mutation could also enable the use of other routine PET radionuclides with a shorter half-life for immuno-PET, like Copper-64 (t_1/2_ = 12.7 h). For Fluorine-18 (t_1/2_ = 109 min) and Gallium-68 (t_1/2_ = 68 min), the clearance rates are probably still too low.

## Conclusion

[^89^Zr]Zr-Adu^H310A^-8D3, a bispecific aducanumab brain shuttle antibody with the well-known H310A mutation showed faster blood clearance, and earlier differentiation of amyloid-beta-related brain uptake compared to the non-mutated version, [^89^Zr]Zr-Adu-8D3. However, no overall beneficial effects of the mutation were observed for potential use of the tracer for brain imaging.

## Supplementary Information

Below is the link to the electronic supplementary material.

Supplementary file1 (DOCX 2.91 MB)

## Data Availability

The datasets generated during and/or analyzed during the current study are available from the corresponding author upon reasonable request.

## Abbreviations

AD: Alzheimer's disease
Adu: Anti-amyloid-beta aducanumab derivate
BBB: Blood-brain barrier
CHO-S: Chinese hamster ovary cells in suspension
CNS: Central-nervous-system
DFO: Desferrioxamine
ELISA: Enzyme-linked immunosorbent assay
FACS: Fluorescence-activated cell sorting
Fc: Fragment crystallizable
FcRn: Neonatal fragment crystallizable receptor FDA: u.s. food and drug administration
HIV: Human immunodeficiency virus
ID: Injected dose
IgG: Immunoglobulin G
immuno-PET: Immuno-positron emission tomography
i.v.: Intravenous
KIH: Knobs-into-holes
mAb: Monoclonal antibody
mTfR1: Murine transferrin receptor1
p.i.: Post injection
RMT: Receptor-mediated transcytosis
scFab: Single chain fab fragment
scFv: Single chain variable fragment
SD: Standard deviation
SE-HPLC: Size-exclusion high-performance liquid chromatography
TG: Transgenic
WT: Wild type
