# Supplementary material for: Investigation of the Impact of the H310A FcRn Region Mutation on 89Zr-Immuno-PET Brain Imaging with a BBB-Shuttle Anti‑Amyloid Beta Antibody
Source: Mol Imaging Biol. 2024 Aug 2;26(5):823–34. doi: 10.1007/s11307-024-01931-z (PMC11436416; doi:10.1007/s11307-024-01931-z)
Supplement: Supplementary file 1 — Supplementary file1 (DOCX 2.91 MB) [file 11307_2024_1931_MOESM1_ESM.docx]

**Investigation of the impact of the H310A FcRn region mutation on ^89^Zr-immuno-PET brain imaging with a brain penetrant anti‑amyloid beta antibody.**

Thomas E. Wuensche^1^*, Natascha Stergiou^1^*, Iris Mes^1^, Mariska Verlaan^1^, Esther J. M. Kooijman^1^, Albert D. Windhorst^1,2^, Allan Jensen^3^, Ayodeji A. Asuni^3^, Benny Bang-Andersen^3^, Guus A. M. S. Dongen^1,2^, , Danielle J. Vugts^1,2#^, Wissam Beaino^1,2#^ *^1^ Amsterdam UMC location Vrije Universiteit Amsterdam, dept Radiology & Nuclear Medicine, De Boelelaan 1117, Amsterdam, The Netherlands*

*^2^ Amsterdam Neuroscience, Brain imaging, Amsterdam, The Netherlands
^3^ H. Lundbeck A/S, Ottiliavej 9, 2500 Valby, Denmark*

**Authors contributed equally*

*^#^Shared senior authorship*

*Corresponding authors:*

*Thomas E. Wuensche: t.wunsche@amsterdamumc.nl*

*Danielle J. Vugts: d.vugts@amsterdamumc.nl*

*Wissam Beaino: w.beaino@amsterdamumc.nl*

Table S1: Summary of radiolabeling results and in vivo study groups.

| *Antibody* | *RCY*  *(%)* | *RCP*  *(%)* | *Mice* | *Age & number of mice* | *Average weight (g)* | *Specific activity (MBq/µg)* | *Molar activity*  *(GBq/µmol)* | *Injected dose (MBq)* |
| --- | --- | --- | --- | --- | --- | --- | --- | --- |
| *[^89^Zr]Zr-Adu^H310A^-8D3* | *94.5* | *≥98* | *APP/PS1 TG female* | *n=5, 10 months* | *29.4 ±4.7* | *0.16* | *31.3^a^* | *5.0 ±0.1* |
|  |  |  | C57BL/6 *WT control female* | *n=5, 10 months* | *28.5 ±2.4* |  |  | *5.0 ±0.4* |
| *[^89^Zr]Zr-*  *Adu-8D3* | *82.0* | *≥98* | *APP/PS1 TG male* | *n=3, 12 months* | *46.6 ±6.0* | *0.20* | *39.1^b^* | *6.7 ±0.2* |
| *[^89^Zr]Zr-B12^H310A^-8D3* | *86.9* | *≥97* | *APP/PS1 TG female* | *n=5, 10 months* | *26.1 ±4.2* | *0.15* | *29.6^c^* | *4.8 ±0.3* |

^a^M_w(theoretical)_ = 195536 Da ^b^M_w(theoretical)_ = 195668 Da ^c^M_w(theoretical)_ = 197528 Da

***In vitro* binding**

*Antigen binding - amyloid-beta Var24-peptide ELISA*

The influence of mAb modification and radiolabeling on the binding to amyloid-beta was evaluated as previously reported.[1] ELISA was performed on [^89^Zr]Zr-Adu-8D3 and [^89^Zr]Zr-Adu^H310A^-8D3 constructs with [^89^Zr]Zr-B12^H310A^-8D3 as the negative control. 96 well polystyrene plates (flat bottom, half area, high binding, Costar®) were coated with 50 µL/well of 100 ng/mL amyloid-beta Var24-peptide (provided by Lundbeck A/S) in 0.1 M borate buffer (pH = 11) at 4 °C overnight. The coating solution was disposed, and the wells were blocked using 150 µL/well of 2% BSA in PBS while shaking the plate at room temperature (RT) and 600 rpm for 2 hours. After disposing the blocking solution, incubation was performed in duplicates with a serial dilution of antibody in incubation buffer (0.1% BSA in PBS + 0.05% Tween-20) at RT and 600 rpm for 1 hour (12 points horizontally, 1:2 dilutions, 50 µL/well). Subsequently, the supernatant was removed, and the wells were washed three times with 0.1% Tween-20 in PBS and incubated with 50 µL/well of goat anti-human IgG (H+L) cross-absorbed-HRP secondary antibody (Invitrogen, 31412; 0.8 µg/mL in incubation buffer), at RT and 600 rpm for 1 hour. After incubation, the solution was removed, and the wells were washed four times with 0.1% Tween-20 in PBS and one time with deionized water (dH_2_O) before adding 50 µL/well of 3,3',5,5'-Tetramethylbenzidine (TMB) substrate for 5 to 10 minutes without shaking and under dark conditions. The enzymatic reaction was stopped with 50 µL/well of 0.5 M HCl solution, and the absorbance at 450 nm was measured immediately with a microplate reader (TriStar^2^ multimode reader LB 942, Berthold Technologies). The absorbance of 0 ng/mL primary antibody was used as the background value and subtracted from the other values. Values are given in relative absorbance by dividing all values by the highest absorbance value of each row. In the case of the negative control, the highest average absorbance of the unmodified Adu-8D3 or Adu^H310A^-8D3 was used.


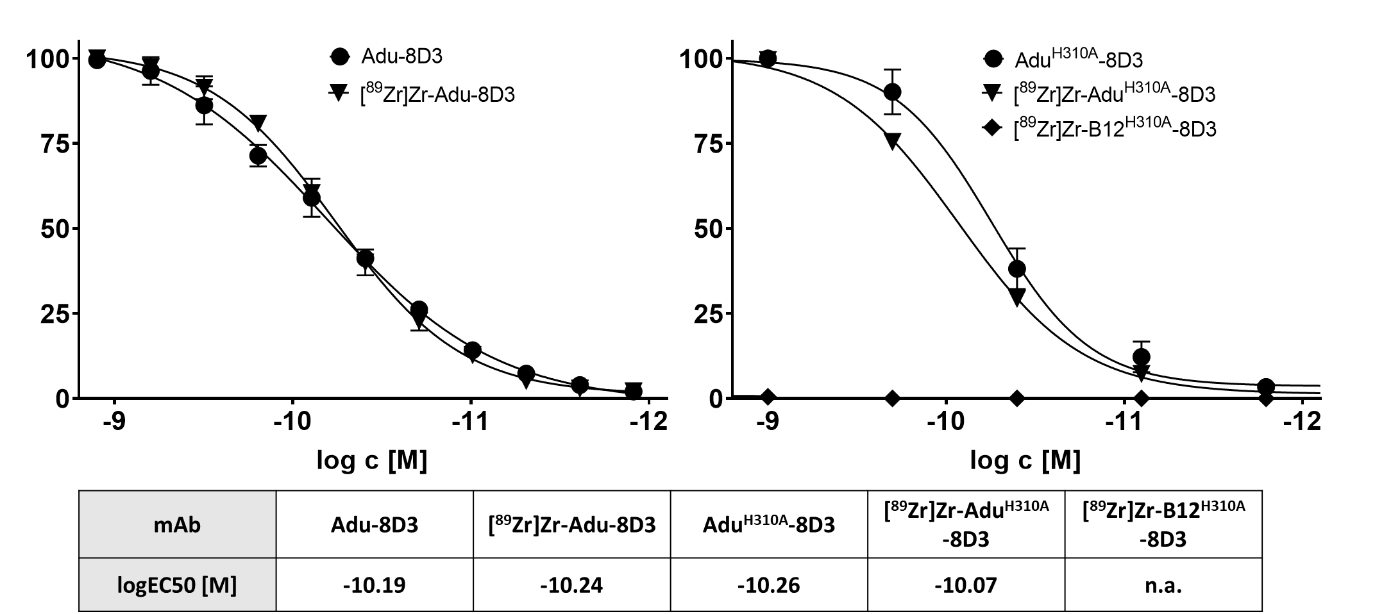


Figure S1: Indirect amyloid-beta Var24 (amyloid-beta vaccine construct consisting of three amyloid-beta 1-12 peptides) ELISA with [^89^Zr]Zr-Adu^H310^-8D3, [^89^Zr]Zr-Adu-8D3, unlabeled Adu-8D3, Adu^H310A^-8D3 and [^89^Zr]Zr-B12^H310A^-8D3 as a negative control. EC50 values were calculated by using the normalized OD at 450 nm values and the following formula [Agonist] vs. [response] – [Variable slope], GraphPad Prism 9.3.1.

*Antigen binding – mTfR1 transfected CHO-S cells FACS analysis*

The binding of all modified mAbs to murine TfR1 was assessed by FACS, as previously reported.[1] Stably transfected CHO-S cell lines with mTfR1 and mock-transfected CHO-S cells were cultured in ExpiCHO Expression Medium (Gibco, cat# A29100-01) with 1% anti-clumping agent (Gibco, cat# 0010057AE) at a cell concentration between 1x10^5 and 2x10^6 viable cells/mL in shaker flasks at 37 °C, 5% CO_2_. The mTfR1 high-expression cells were grown under selection pressure (12 mg/mL Puromycin dihydrochloride (Merck, cat# P9620-10ML)). To prepare the mTfR1 transfected and mock-transfected CHO-S cells for FACS, cells were harvested, washed 3 times with cold PBS (spin 300xg for 5 min), counted (viable cell count, Cedex Hires) and adjusted to 5x10^6 cells/mL in cold PBS. 100 µL of cells were transferred into a 96-well-multi dish (U bottom) plate (0.5x10^6 cells/well), spun down for 5 min at 400xg at 4 °C, and the supernatant was discarded. The cells were stained with live/dead cell stain (L34963, 405nm, Invitrogen). Therefore, a mastermix of 0.1 µL dye in 100 µL PBS w/o Mg^2+^ and Ca^2+^ per sample was prepared. The cells were incubated on ice for 15 min in the dark and washed 3 times by adding a total of 180 mL cold FACS buffer (250 mL PBS w/o Ca^2+^ and Mg^2+^ + 2 mM EDTA (1 mL 0.5 M), 2% normal goat serum) per well and centrifuged for 5 min with 350xg at 4 °C. The cells were blocked by adding 50 µL 10% normal goat serum containing buffer/well (1xPBS w/o Ca^2+^ and Mg^2+^ + 2 mM EDTA (60 µL 0.5M, 10% normal goat serum), mixed well, and incubated on ice in the dark for 15 min. After that, the plate was spun down, and the supernatant was discarded. The non-modified and modified mAbs (Adu, Adu-8D3, B12-8D3) were added in a total volume of 85 µL with a concentration of 1 nM/2x10^5 cells, incubated for 20 min on ice in the dark, 3 times washed as described above with FACS buffer. Secondary antibody (goat anti-human IgG, Jackson, cat. # 109-605-008) diluted 1:400 was added in a total volume of 100 µL in FACS buffer was added to wells, incubated for 20 min on ice in the dark, and washed 3 times with FACS buffer. The cells were fixed with 100 µL of 4% PFA (BD Fixation buffer, cat# 554655) and incubated for 15 min on ice in the dark. Cells were washed 3 times with FACS buffer and resuspended in 180 µL FACS analysis buffer (1xPBS + 2 mM EDTA, 1% BSA (0.1 g/10 mL) (IgG/protease-free, Jackson # 001-000-162, lot# 138456)). Samples were kept on ice in the dark until FACS analysis using a NovoCyte Quanteon. Cells were gated for live, single cells, and the mean fluorescence intensity (MFI AF647) and the percentage of binding were determined for each mAb and modified mAb in comparison to the unspecific binding of the secondary antibody conjugated with AF647 (FlowJo 10 software).

Table S2: Flow cytometry analysis of the mAb-8D3 constructs (±H310A mutation, ±DFO* modification) to validate binding to mTfR1. Unmodified and non-mutated Adu was used as negative controls.

| **mAb** | **MFI (AF647)** | **%-binding** |
| --- | --- | --- |
| Adu [2] | 189 | 0 |
| Adu-8D3 [2] | 54413 | 97 |
| DFO*-Adu-8D3 | 40752 | 100 |
| Adu^H310A^-8D3 | 50226 | 100 |
| DFO*-Adu^H310A^-8D3 | 37899 | 98 |
| B12^H310A^-8D3 | 29077 | 98 |
| DFO*-B12^H310A^-8D3 | 19168 | 94 |


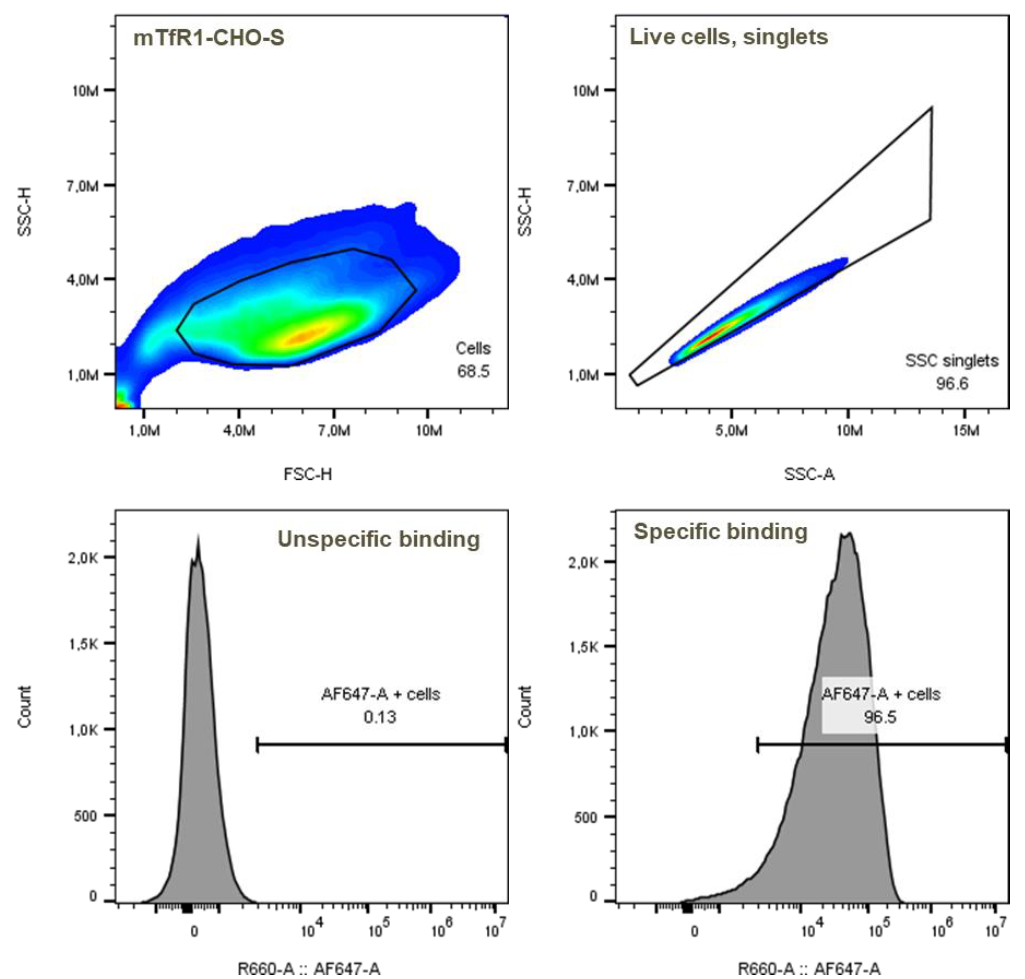


Figure S2: An example figure for the used FACS gating strategy was taken from Wuensche et al..[1] Cells were gated to remove debris (top left) for live and single cells (top right), and the % of binding (% of positive cells for the antibody tested that is labeled with AF647). The percentage of binding was determined for each mAb construct and compared with the unspecific binding of the secondary antibody conjugated with AF647 (bottom left and right) via FlowJo 10 software.

**Radiochemical purity**

Radioimmunoconjugates were checked for radiochemical purity by spin filter analysis following a described procedure.[3] 1 µL of product was diluted to 100 µL with wash buffer (formulation buffer + 5% DMSO) and pipetted onto a 30 kDa cut-off spin filter (Ultracel YM-30, regenerated cellulose, 30 kDa cut-off, Merck Millipore), which was subsequently centrifuged at 14000 rpm for 7 min (Eppendorf 5430). The filter was then washed with 100 µL of the wash buffer and spun again for 7 min at 14000 rpm before being rewashed with 100 µL buffer and spun down again at the same settings. Subsequently, the filter and combined filtrate were counted separately in a gamma counter (LKB Wallac Gamma Counter, model 1282 Compugamma CS). Radiochemical purity was determined by calculating the ratio of counts on the filter (with background subtracted) to the total number of counts of the filtrate plus the counts on the filter (with background subtracted).

***SE-HPLC chromatograms of mAbs***

Protein concentration was determined by size-exclusion high-performance liquid chromatography (SE-HPLC). In short, a Jasco or Shimadzu HPLC system was equipped with a Superdex® 200 Increase 10/300 GL (30 cm × 10 mm, 8.6 μm) size exclusion column (GE Healthcare Life Sciences) and a guard column using 0.05 M phosphate buffer/0.15 M NaCl/0.01 M NaN_3_ (pH = 6.7) as mobile phase with a run time of 40 min at 0.75 mL/min. Antibody concentration was assessed using the areas under the curve on the UV channel at 280 nm. The concentration was determined against a calibration curve of the unlabeled compound.


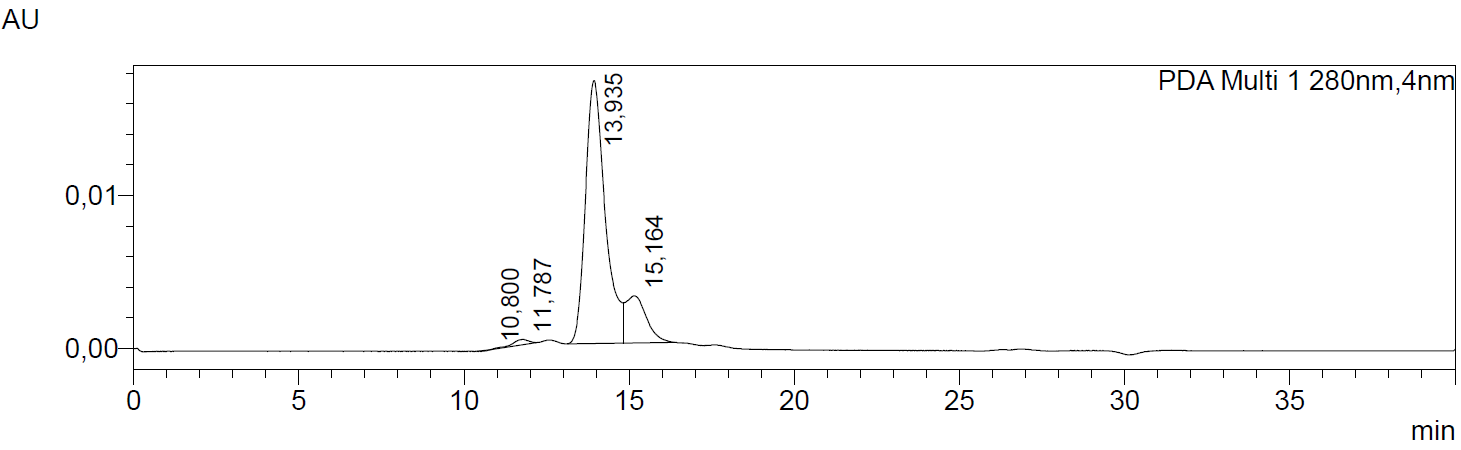


Figure S3: SE-HPLC chromatogram (UV absorption at 280 nm) of Adu^H310A^-8D3.


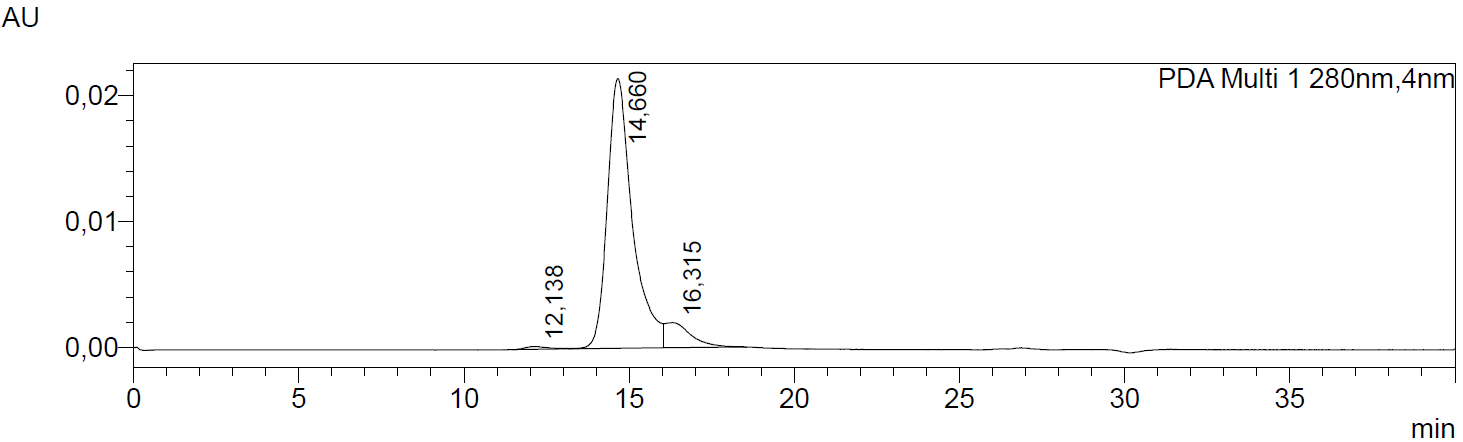


Figure S4: SE-HPLC chromatogram (UV absorption at 280 nm) of B12^H310A^-8D3.


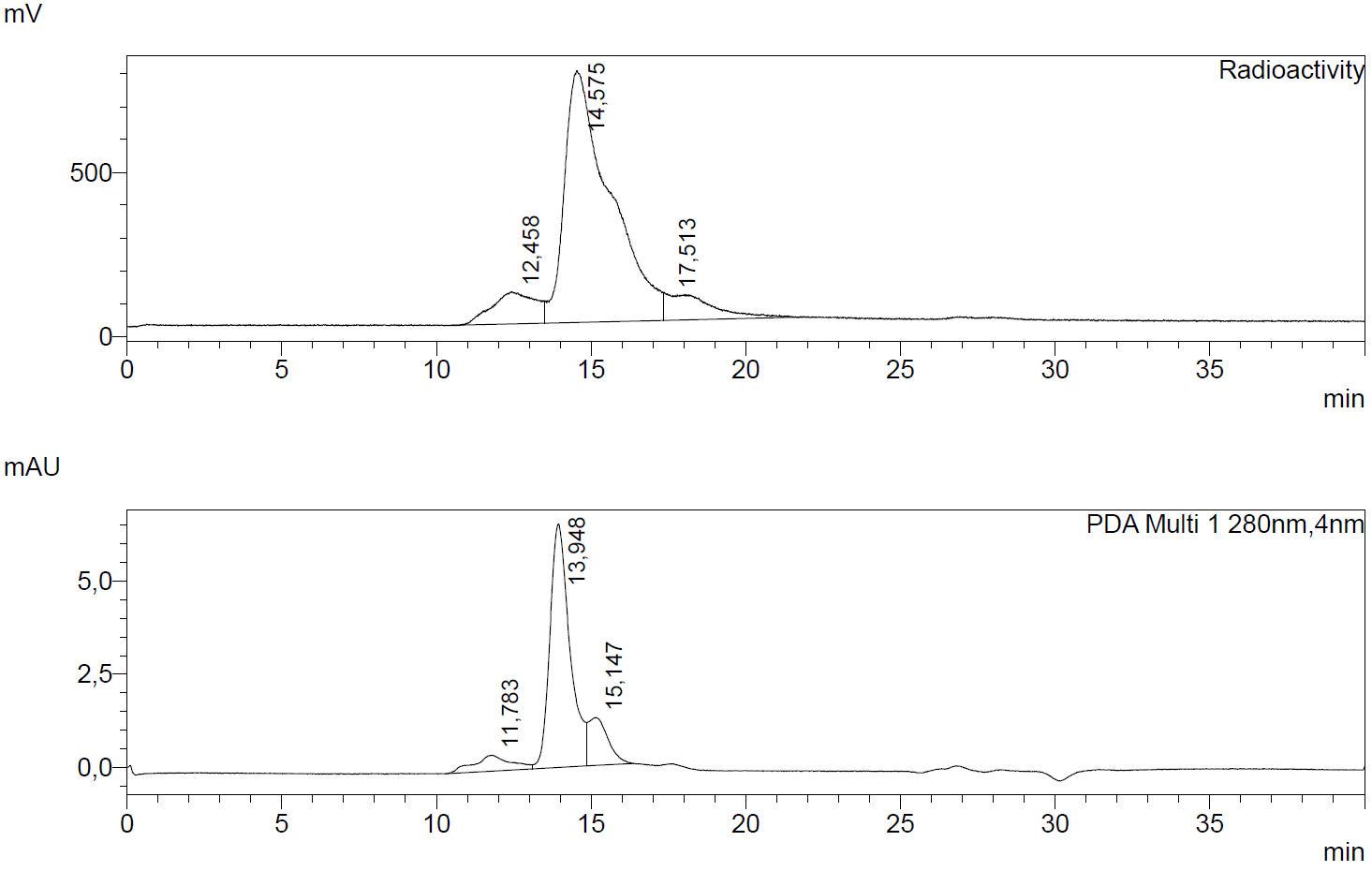


Figure S5: SE-HPLC chromatogram of [^89^Zr]Zr-Adu^H310A^-8D3 after formulation (unlabeled Adu^H310A^-8D3 and buffer added). Top panel: radiodetector, bottom panel: UV absorption at 280 nm.


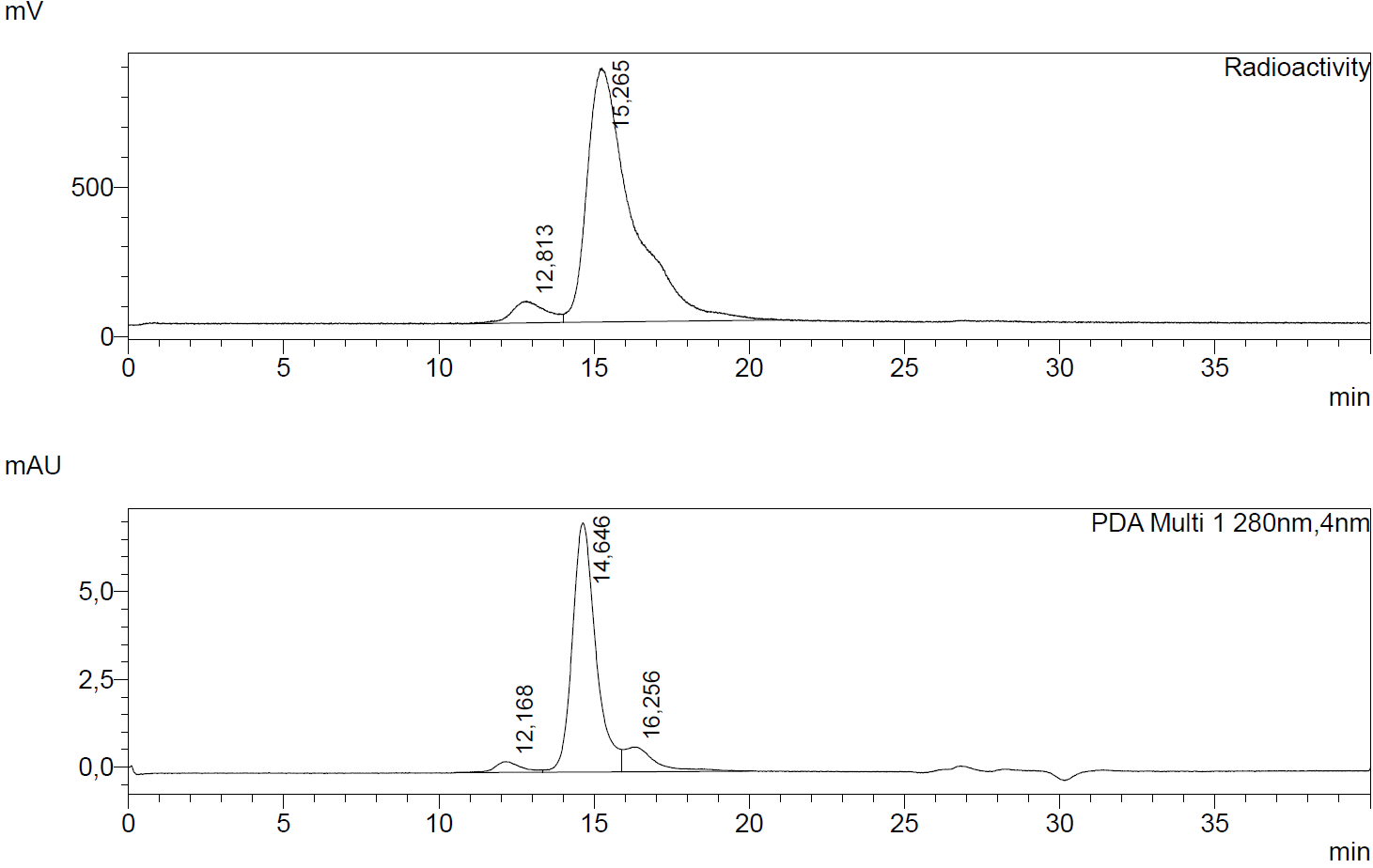


Figure S6: SE-HPLC chromatogram of [^89^Zr]Zr-B12^H310A^-8D3 after formulation (unlabeled B12^H310A^-8D3 and buffer added). Top panel: radiodetector, bottom panel: UV absorption at 280 nm

**Determination of chelator-to-mAb ratio**

A SE-MS approach to analyze the chelator-to-mAb ratio, as reported by Sijbrandi et al., was again not possible for the DFO*-NCS and DFO-NCS modified mAb samples due to the thiourea bond in those conjugates as previously reported. [1, 4, 5] We also considered titration methods as reported before. However, this method could not be performed due to the large amounts of antibodies needed, which makes it more suitable for commercially available antibodies.[6, 7] Due to these constraints, only calculations to approximate the chelator-to-mAb ratio are possible. Considering the molar activity of the utilized ^89^Zr (20-30 MBq/nmol) and the obtained yields for the NCS modifications involving 150-250 MBq ^89^Zr and 500 μg (2.5 nmol) protein, it can be assumed that a chelator-to-mAb ratio ranging from 2:1 to 5:1 was achieved.

**Blood kinetics of all groups: non-logarithmic representation**

Figure S7: Blood kinetics of [^89^Zr]Zr-Adu^H310A^-8D3 in APP/PS1 TG and WT control mice as well as blood kinetics of [^89^Zr]Zr-Adu-8D3 and [^89^Zr]Zr-B12^H310A^-8D3 in APP/PS1 TG mice. 10-12 months old APP/PS1 TG or WT control mice were injected with 1 mg/kg radioimmunoconjugates, and blood sampling was performed up to 72 hrs p.i..

**B12^H310A^-8D3 in APP/PS1 TG mice**


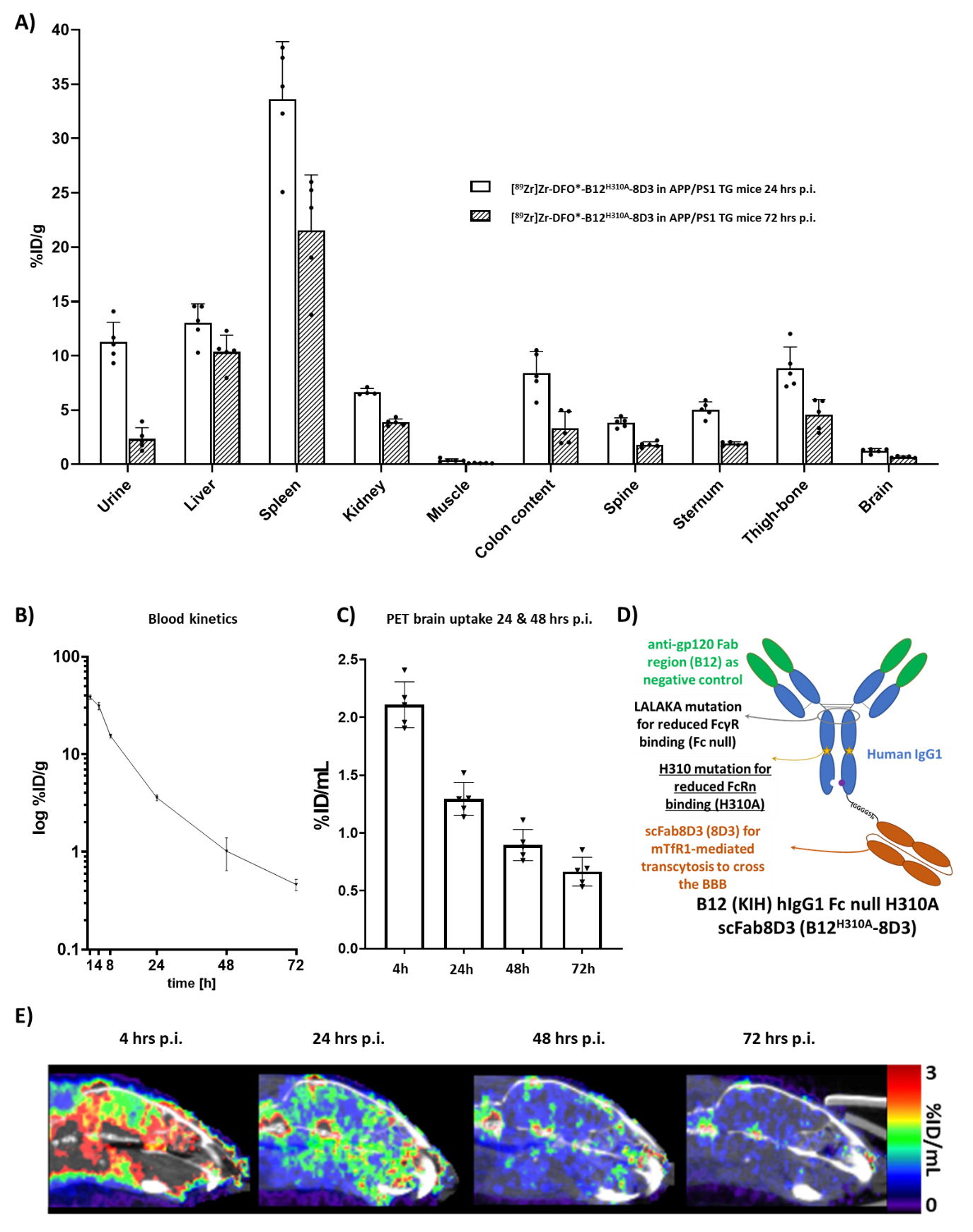


Figure S8: Blood kinetics, PET brain uptake, ex vivo biodistribution, ex vivo autoradiography, and immunofluorescence of [^89^Zr]Zr-B12^H310A^-8D3 in APP/PS1 TG. 10 months old APP/PS1 TG mice were injected with 1 mg/kg radioimmunoconjugates, and blood sampling was performed up to 72 hrs p.i.. Ex vivo biodistribution analysis was performed at 24 and 72 hrs p.i.. A) Ex vivo uptake of selected organs. B) Blood kinetics. C) PET brain quantification D) Structure of B12^H310A^-8D3 E) One sagittal representative PET/CT image is shown per group.

**Immunofluorescence staining of Adu^H310A^-8D3 in APP/PS1 TG mice: magnified images**


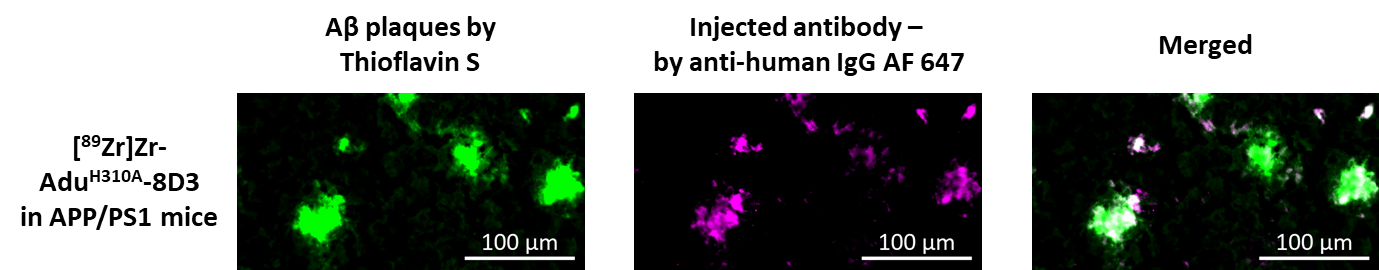


Figure S9: Immunofluorescence analysis of 20 μm brain cryo-sections of APP/PS1 TG mice at 72 hrs p.i.. 10 month old APP/PS1 TG mice were injected with 1 mg/kg radiolabeled antibody. The sections were stained with 0.125% Thioflavin S (green) and AF647-goat anti-human IgG (1:1000, purple) to detect the injected antibody. The images of each separate and merged channel are shown; the overlay of the two signals appears in white.

**Whole body PET images – 72 hours p.i.**


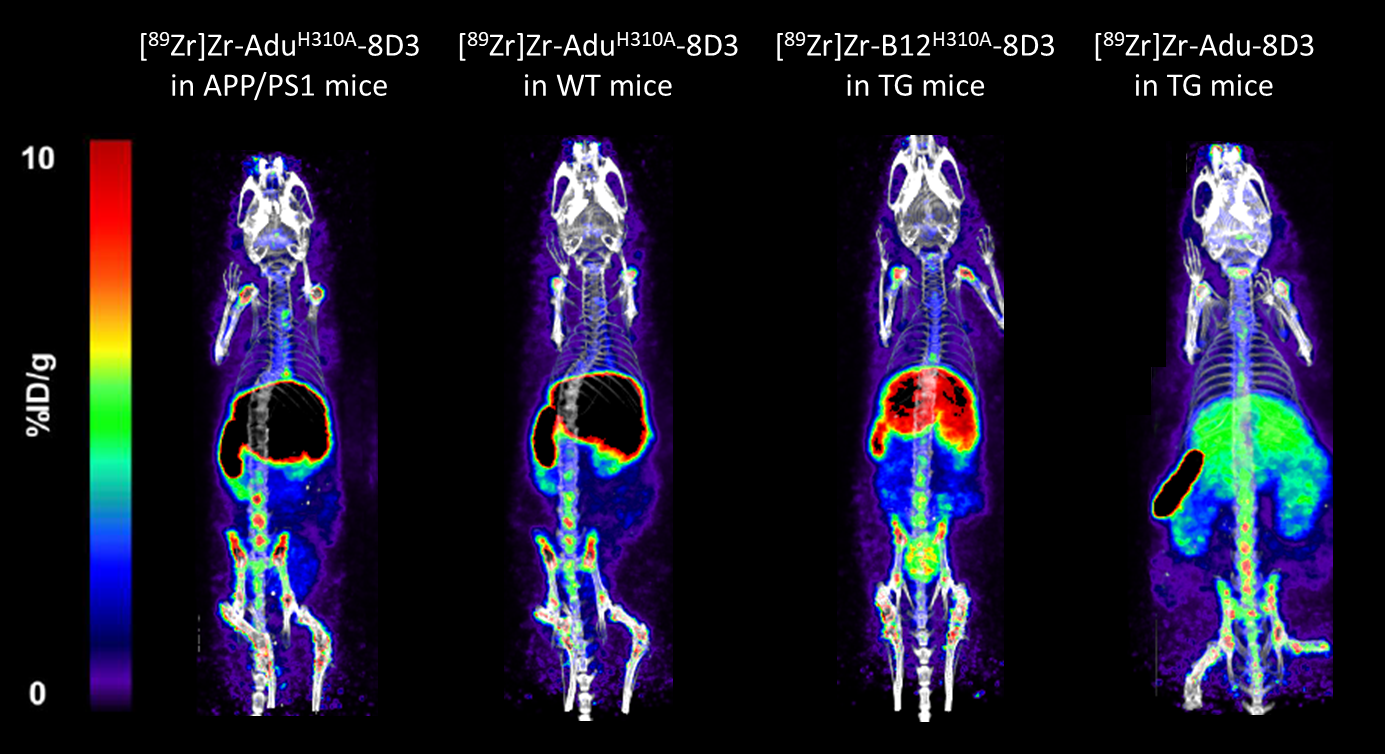


Figure S10: Representative whole-body PET/CT MIP images for all in vivo study groups 72 hrs p.i., injected with ~30 µg of radioimmunoconjugate.

**Pet brain uptake: Region of interest**

**
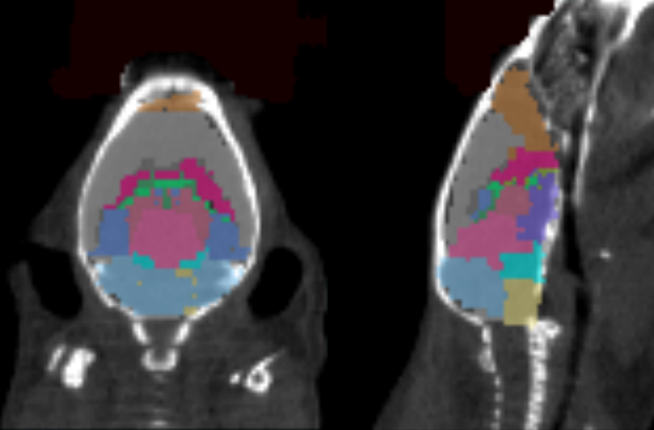
**

Figure S11: Example region of interest (ROI) that was applied using the VivoQuant-integrated brain atlas tool to quantify and analyze the PET brain uptake. The reported PET brain uptake values were derived from the average uptake values of the entire atlas.

***Ex vivo* biodistribution data**

Table S3: Ex vivo Biodistribution of [^89^Zr]Zr-mAb^H310A^ conjugates in 10-12 month-old APP/PS1 TG or 10 month old WT control mice, 24 hrs after administration of 1 mg/kg of conjugate. Results are expressed as mean (%ID/g) ± sd (n=4-5 mice per group).

| **Organ** | **Adu^H310A^-8D3 TG** | **Adu^H310A^-8D3 WT** | **B12^H310A^-8D3 TG** |
| --- | --- | --- | --- |
| Blood | 1.52 ± 0.21 | 1.84 ± 0.48 | 3.65 ± 0.4 |
| Urine | 8.03 ± 2.48 | 10.91 ± 2.18 | 11.26 ± 1.81 |
| Skin | 0.92 ± 0.23 | 1.13 ± 0.05 | 1.24 ± 0.26 |
| Bladder | 1.8 ± 0.49 | 1.51 ± 0.08 | 2.41 ± 0.46 |
| Sternum | 2.84 ± 0.74 | 3.12 ± 0.40 | 5.03 ± 0.72 |
| Heart | 1.9 ± 0.19 | 1.86 ± 0.17 | 2.61 ± 0.19 |
| Lung | 1.83 ± 0.19 | 2.04 ± 0.22 | 2.51 ± 0.34 |
| Liver | 18.25 ± 3.45 | 20.38 ± 2.99 | 13.01 ± 1.77 |
| Pancreas | 0.4 ± 0.06 | 0.38 ± 0.02 | 0.79 ± 0.7 |
| Spleen | 32.31 ± 3.97 | 33.94 ± 2.13 | 33.6 ± 5.32 |
| Kidney L | 8.17 ± 0.73 | 9.51 ± 0.75 | 8.62 ± 4.39 |
| Muscle | 0.22 ± 0.05 | 0.24 ± 0.02 | 0.38 ± 0.14 |
| Thigh-bone | 6.23 ± 0.99 | 7.2 ± 0.88 | 8.86 ± 1.95 |
| Colon | 1.88 ± 0.27 | 1.79 ± 0.17 | 2.27 ± 0.68 |
| Colon content | 4.14 ± 0.53 | 5.63 ± 1.31 | 8.42 ± 1.96 |
| Ileum | 8.75 ± 3.1 | 9.03 ± 1.54 | 10.04 ± 2.87 |
| Ileum content | 4.08 ± 0.8 | 2.71 ± 0.71 | 3.98 ± 1.15 |
| Stomach | 1.68 ± 0.4 | 1.79 ± 0.14 | 3.13 ± 1.25 |
| Stomach content | 0.85 ± 0.55 | 1.49 ± 0.38 | 1.41 ± 1.08 |
| Tail | 2.41 ± 1.48 | 2.1± 1.23 | 1.60 ± 0.32 |
| Spine | 2.35 ± 0.43 | 2.84 ± 0.5 | 3.84 ± 0.45 |
| Brain hemisphere | 1.03 ± 0.14 | 0.84 ± 0.16 | 1.24 ± 0.21 |

Table S4: Ex vivo Biodistribution of [^89^Zr]Zr-mAb H310A conjugates in 10 months old APP/PS1 transgenic or 10 months old WT littermates, 72 hrs after administration of 1 mg/kg of conjugate. Results are expressed as mean (%ID/g) ± sd (n=5 mice per group).

| **Organ** | **Adu^H310A^-8D3 TG** | **Adu^H310A^-8D3 WT** | **B12^H310A^-8D3 TG** |
| --- | --- | --- | --- |
| Blood | 0.27 ± 0.02 | 0.22 ± 0.02 | 0.46 ± 0.06 |
| Urine | 2.75 ± 1.06 | 2.25 ± 1.2 | 2.34 ± 1.04 |
| Skin | 0.31 ± 0.16 | 0.44 ± 0.1 | 0.37 ± 0.09 |
| Bladder | 0.84 ± 0.11 | 0.87 ± 0.18 | 0.86 ± 0.18 |
| Sternum | 1.94 ± 0.49 | 1.82 ± 0.38 | 1.91 ± 0.17 |
| Heart | 0.91 ± 0.07 | 0.8 ± 0.06 | 0.97 ± 0.12 |
| Lung | 0.76 ± 0.19 | 0.71 ± 0.09 | 1.04 ± 0.59 |
| Liver | 15.69 ± 0.68 | 16.12 ± 0.52 | 10.35 ± 1.55 |
| Pancreas | 0.21 ± 0.04 | 0.19 ± 0.02 | 0.2 ± 0.06 |
| Spleen | 36.45 ± 4.59 | 29.88 ± 5.24 | 21.54 ± 5.11 |
| Kidney L | 6.49 ± 0.97 | 5.82 ± 0.24 | 3.86 ± 0.33 |
| Muscle | 0.11 ± 0.02 | 0.11 ± 0.02 | 0.12 ± 0.02 |
| Thigh-bone | 4.63 ± 0.68 | 3.96 ± 0.53 | 4.57 ± 1.39 |
| Colon | 0.94 ± 0.24 | 1.05 ± 0.1 | 1.04 ± 0.21 |
| Colon content | 1.74 ± 0.37 | 1.27 ± 0.5 | 3.35 ± 1.5 |
| Ileum | 2.54 ± 0.25 | 2.55 ± 0.26 | 3.07 ± 0.3 |
| Ileum content | 2.24 ± 0.49 | 2.14 ± 0.23 | 2.7 ± 0.23 |
| Stomach | 0.54 ± 0.05 | 0.54 ± 0.08 | 0.55 ± 0.06 |
| Stomach content | 0.22 ± 0.09 | 0.32 ± 0.04 | 0.38 ± 0.1 |
| Tail | 1.74 ± 1.07 | 1.02 ± 0.3 | 1.45 ± 1.16 |
| Spine | 1.64 ± 0.2 | 1.66 ± 0.21 | 1.82 ± 0.26 |
| Brain hemisphere | 0.95 ± 0.07 | 0.54 ± 0.03 | 0.67 ± 0.08 |

**References**

1. Wuensche TE, Stergiou N, Mes I, et al (2022) Advancing 89Zr-immuno-PET in neuroscience with a bispecific anti-amyloid-beta monoclonal antibody – the choice of chelator is essential. Theranostics 12:7067–7079. https://doi.org/10.7150/thno.73509

2. Stergiou N, Wuensche TE, Schreurs M, et al (2023) Application of 89Zr-DFO*-immuno-PET to assess improved target engagement of a bispecific anti-amyloid-ß monoclonal antibody. Eur J Nucl Med Mol Imaging 1306–1317. https://doi.org/10.1007/s00259-023-06109-3

3. Vugts DJ, Klaver C, Sewing C, et al (2017) Comparison of the octadentate bifunctional chelator DFO*-pPhe-NCS and the clinically used hexadentate bifunctional chelator DFO-pPhe-NCS for 89Zr-immuno-PET. Eur J Nucl Med Mol Imaging 44:286–295. https://doi.org/10.1007/s00259-016-3499-x

4. Sijbrandi NJ, Merkul E, Muns JA, et al (2017) A Novel Platinum(II)-Based Bifunctional ADC Linker Benchmarked Using Zr-Desferal and Auristatin F Conjugated Trastuzumab. Cancer Res 77:257–267

5. Chomet M, Schreurs M, Bolijn MJ, et al (2021) Head-to-head comparison of DFO* and DFO chelators: selection of the best candidate for clinical 89Zr-immuno-PET. Eur J Nucl Med Mol Imaging 48:694–707. https://doi.org/10.1007/s00259-020-05002-7

6. Deri MA, Ponnala S, Kozlowski P, et al (2015) P-SCN-Bn-HOPO: A Superior Bifunctional Chelator for 89Zr ImmunoPET. Bioconjug Chem 26:2579–2591. https://doi.org/10.1021/acs.bioconjchem.5b00572

7. Vugts DJ, Klaver C, Sewing C, et al (2017) Comparison of the octadentate bifunctional chelator DFO*-pPhe-NCS and the clinically used hexadentate bifunctional chelator DFO-pPhe-NCS for 89Zr-immuno-PET. Eur J Nucl Med Mol Imaging 44:286–295. https://doi.org/10.1007/s00259-016-3499-x
